# DRREP: deep ridge regressed epitope predictor

**DOI:** 10.1186/s12864-017-4024-8

**Published:** 2017-10-03

**Authors:** Gene Sher, Degui Zhi, Shaojie Zhang

**Affiliations:** 1 Department of Computer Science, University of Central Florida, Orlando, FL USA; 2 School of Biomedical Informatics, University of Texas Health Science Center at Houston, Houston, TX USA

**Keywords:** Epitope prediction, Deep network, Neural network, Analytical learning, Linear epitope, Continuous epitope, Convolutional network, String kernel

## Abstract

**Introduction:**

The ability to predict epitopes plays an enormous role in vaccine development in terms of our ability to zero in on where to do a more thorough in-vivo analysis of the protein in question. Though for the past decade there have been numerous advancements and improvements in epitope prediction, on average the best benchmark prediction accuracies are still only around 60%. New machine learning algorithms have arisen within the domain of deep learning, text mining, and convolutional networks. This paper presents a novel analytically trained and string kernel using deep neural network, which is tailored for continuous epitope prediction, called: Deep Ridge Regressed Epitope Predictor (DRREP).

**Results:**

DRREP was tested on long protein sequences from the following datasets: SARS, Pellequer, HIV, AntiJen, and SEQ194. DRREP was compared to numerous state of the art epitope predictors, including the most recently published predictors called LBtope and DMNLBE. Using area under ROC curve (AUC), DRREP achieved a performance improvement over the best performing predictors on SARS (13.7%), HIV (8.9%), Pellequer (1.5%), and SEQ194 (3.1%), with its performance being matched only on the AntiJen dataset, by the LBtope predictor, where both DRREP and LBtope achieved an AUC of 0.702.

**Conclusion:**

DRREP is an analytically trained deep neural network, thus capable of learning in a single step through regression. By combining the features of deep learning, string kernels, and convolutional networks, the system is able to perform residue-by-residue prediction of continues epitopes with higher accuracy than the current state of the art predictors.

## Introduction

Early methods for vaccine development were based on the micro-organisms themselves [1]. In contrast, the promise of successful subunit vaccines (epitope-driven vaccine) comes from antigens [2]. Our ability to design subunit vaccines depends on our ability to find good vaccine targets on the foreign object in question, it depends on our ability to find epitopes. There are numerous experimental techniques for B-cell epitope mapping [3], but doing this search experimentally, searching exhaustively by brute force, is an extremely time consuming endeavour. Thus, computational approaches are employed, and are the primary subject domain of computational vaccinology. Epitope prediction, the ability to predict with some probability whether a particular amino acid belongs to an epitope, can guide our experimental based search and save us a significant amount of time.

B-Cell epitopes are antigenic residues that B Lymphocytes bind to. These antigenic determinants can be either continuous, or conformational. Continuous epitopes, also known as linear epitopes, are formed by continuous sequences of residues. The majority of epitopes (90%) are within the conformational class [4], which are the result of 3D interaction of protein residues in close spatial proximity, but discontinuous on the actual protein sequence. Predicting both of these epitope types is a computationally difficult, and because conformational epitopes can be considered as clusters or spatially joined continuous epitopes, the prediction of continuous eptopes is an essential step for both problems. Finally, because there is a substantially larger amount of epitope sequence based data than there is structural, protein sequence based (as opposed to structure based) prediction is a much more feasible problem at this time.

In this work we present a sequence based continuous epitope predictor called DRREP (Deep Ridge Regressed Epitope Predictor). Our *linear B-Cell epitope* predictor is based on a deep neural network (DNN) [5], which utilizes a string mismatch function based first hidden layer, a second normalization pooling layer, an analytically computed third hidden layer, followed by another non-linear pooling layer, and a final fifth layer composed of a single threshold neuron. Our intuition is that because there is structure within protein sequences, and because we are dealing with sequences composed of characters, these structures and patterns are based on the k-mers within the sequences. Thus, a way to find and extract them, is through the use of string based activation functions, similar to methods applied in text mining. Because we do not know ahead of time the actual structures, lengths, and patterns of these k-mers, one way to solve the problem of exposing them is to generate a large number of our own random k-mer patterns, tiling the sequence with them, and counting how many, and which of our generated k-mers match the k-mers within the sequence being analysed. This k-mer tiling method extrapolates the protein sequence into a large feature space, which we can then cluster, separate, and classify through regression. In DRREP, we perform this regression step using the Moore-Penrose generalized inverse [6].

## Background

The first linear epitope prediction methods were developed in the 1980s, and were based on propensity scales [7, 8]. These were built up experimentally, and based on the statistical correlation of a physicochemical property of a residue and it belonging to an epitope. Later systems used multiple propensity scales together, these systems include the likes of PEOPLE [9], PREDITOP [10], BEPITOPE [11], and BcePred [12]. A decade later, these propensity scales were coupled with various predictive algorithms, after it was shown that predictions based purely on propensity scales produce results only slightly better than random [13].

Starting in 2006, machine learning algorithms coupled with new types of amino acid sequence encoding methods and propensity scales, began to emerge. The first of such systems was ABCPred [14], based on a recurrent neural network with an input vector of 16 residues using a sparse binary encoding. During the same year, BepiPred [15] was released, and was based on Hidden Markov Models [16] rather than neural networks. The input to BepiPred was based on numerous physicochemical properties and protein secondary structure. In 2007 AAP [17] was released, and was the first predictor using a support vector machine based model, and proposed the use of a new type of antigenicity propensity scale. AAP’s improved performance ushered a new era of epitope predictors based on SVM algorithms.

In 2008, BCPred [18], and later in 2010, FBCPred [19] were published, both using SVM. BCPred and FBCPred demonstrated that predictive improvement can be achieved by using methods developed within the text mining community. These two systems used string kernels [20, 21] and SVM to make their predictions. BCPred operates on a fixed length input sequence window, whereas FBCPred can be applied to variable length input sequences. LEP-LP [22] is an SVM predictor released in 2008, and was based on multiple numerically profiled propensity scales as input.

Due to SVM’s excellent classification performance, the SVM based predictor trend continues to this day. CBTOPE [23] converts residues in the sliding window into a “Composition profile of patterns”, which is a vector of amino acid ratios within the window. BEST [24] epitope predictor uses a 20-mer sliding window and an SVM classifier. COBEpro [25] is another epitope predictor which uses SVM to predict short epitope sub-sequences. All three, CBTOPE, BEST, and COBEpro, can also predict conformational epitopes using a secondary clustering algorithm.

Around the same time, in 2009, EPITOPIA [26] was released. EPITOPIA is based on a naive bayes classifier and is capable of predicting conformational epitopes. It uses structure and sequence based inputs, with the sequence input being based on a sliding window and multiple (14) propensity scales. BaysB [27] is an epitope predictor based on the naive bayes method and an SVM model. BRORacle [28] uses an SVM predictor whose input data is based on sequence features, secondary structure, and physicochemical properties such as solvent accessibility and disorder. LEPS [29] was released in 2011 and is an extension of LEP-LP. LEPS’ SVM based model is used to discard LEP-LP’s less likely candidates, resulting in a more accurate classification. SVMTriP [30] is an SVM based predictor, but for input it uses “Tri-peptide similarity and Propensity scores.”

One of the most recently published epitope predictors is LBtope [31]. LBtope is also based on an SVM model, which is coupled with a nearest neighbour algorithm. In the paper presenting LBtope, Singh et al. notes that until now, most predictors (ABCPred, BCPred, FBCPred, BEST) have been trained on negative datasets composed of random peptides. Furthermore, the training datasets have been small, with a size of roughly 1500 total samples. To solve this problem, Singh et al. composed a new dataset of epitopes and non-epitopes, an order of magnitude larger and using the available data from IEDB [32, 33]. In this LBtope-dataset, the non-epitope sequences were based on confirmed data.

Finally, in 2015 deep learning models began entering the bioinformatics domain. Deep learning, and in particular convolutional deep networks, are currently state of the art in classification. The deep maxout network based model called DMN-LBE [34], was the first deep learning approach which was applied to linear epitope prediction. This predictor used the new LBTope dataset for training and testing, and used 5-fold cross validation. The system’s classification performance was reported to be slightly higher than that of LBTope. Unfortunately, just like LBtope, it was not applied to actual long protein sequences in the published paper.

Taking all of this information into account, in this work we develop a first of its kind, deep analytically learning network using string kernels. Our system, DRREP, due to using string kernels, can be applied to the sequence directly and without any type of pre-processing. Furthermore, DRREP outputs a vector of residue-by-residue scores, rather than scores for a single fixed k-mer window. Thus, DRREP can be used to predict the presence of epitopes in variable length sequences, and applied to entire protein chains. It is a convolutional deep network, with the first layer being a convolutional string kernel, the second an average pooling layer, the third a linear neuron layer, the fourth an average pooling layer, and finally fifth being a single threshold neuron.

## Methods

Majority of the published epitope classifiers are based on Support Vector Machines [2], Neural Networks trained through backpropogation [35, 36], Naive Bayes Classifiers [18], and Propensity Scales [37]. A very limited number of the more obscure methods have also been explored, such as Ant Colony Optimization [38], for example. In the last decade a number of new and innovative classification and regression algorithms have been demonstrated and published, the most promising of which falls into the category of Deep Learning [39]. These types of systems are only now starting to be explored in the bioinformatics, and more concretely, the epitope prediction domain.

In this paper we develop a new epitope classification pipeline called Deep Ridge Regressed Epitope Predictor (DRREP). DRREP is a deep neural network which uses a string mismatch activation function, and is trained using an analytical method based on ridge regression. Because DRREP learns using an analytical method in a single step (going through the data only once), the system learns faster than SVM and other traditional iterative learning methods (exp. those based on error back-propagation).

### Benchmark datasets

There is a need to standardize epitope prediction benchmarking. Different papers discussing their predictors tend to use different test datasets. For example, BCPRED/FBCPRED used a short 193 residue SARS-COV sequence, BepiPred used an HIV dataset composed of 2706 residues, and BEST predictor used a large SEQ194 dataset, composed of 128180 residues. But the Accuracy and AUC achieved by a system on one dataset, can not be compared to the accuracy and AUC achieved by another system on a different dataset. This makes the task of comparing the different epitope predictors a bit difficult, requiring the re-application of the published predictors on some common datasets. Thus, we found the most current test datasets used by other state of the art predictors, and applied our system to those datasets, and when possible (when an epitope prediction server was available), applied the competing predictor to the test datasets it has not been applied to in its original paper. This allowed us to compare the AUC of different predictors on multiple datasets, each with very different epitope densities.

We have chosen to use the following 5 datasets: 1. SARS [40], which is a relatively short (193 residues) sequence, with a high epitope density. 2. HIV [41] dataset on which a number of other predictors have been tested and reported their AUC on, composed of 2706 residues. 3. SEQ194, which is a large dataset derived from BciPep, composed of 194 protein sequences, with a total of 128180 residues, and used as a test dataset by numerous predictors [24]. 4. AntiJen [42] used by BepiPred as a validation dataset. and 5. Pellequer [43], which was used as BepiPred’s training dataset [15].

The SEQ194, HIV, Pellequer, and AntiJen sequences were all calculated by measuring the cross-reactivity between the intact protein and the peptide fragments. AntiJen and SEQ194 have extremely low epitope densities (1.4 and 6.6%, respectively); HIV and Pellequer have an order of magnitude higher epitope densities (37.1 and 37.6%, respectively); and SARS has the highest epitope density of the five datasets (63.7%). Thus, together these 5 datasets represent a realistic test of the classifier that is to be used to search for new epitopes within new protein sequences, covering a wide spectrum of possible epitope densities.

We also wanted a relatively common training dataset that has been used by other predictors, and which did not share any of its epitopes with the test datasets we have selected. We have searched through the literature and found that the BciPep [44] dataset has been utilized as a training dataset by a variety of predictors. The BCPred/FBCPred further pointed out some of the weaknesses within that dataset, producing a variation of it without protein sequence duplicities. A training dataset based on it was also used by the BEST predictor. Thus, given its common use, it represents a good training and validation dataset, and was chosen by us to train and validate DRREP on.

#### Training and validation dataset

DRREP was trained on the BCPred’s 80% homology reduced dataset [45], which is itself a refined, homology reduced BciPep dataset [44]. The BCPred group based their dataset on the BciPep’s shared 1230 unique linear B-Cell epitope dataset, by only keeping the 80% homology reduced subset. Afterwards, any epitope present in the subset that had a length less than 20 amino-acids, was extended/buffered on both sides by random anti-gen sequences from SwissProt [45]. This resulted in a new dataset composed of 1230 linear B-Cell epitopes, each of length *20* or greater. This dataset was further filtered to remove sequences that due to the buffering became too similar. The final dataset was composed of 701, 80% homology reduced sequences, each composed of 20 residues. For this 701 epitope sequence based dataset, non-epitope peptides were then generated by randomly extracting non-eptipe sequences from the SwissProt database, with the final dataset composed of 701 epitopes and 701 non-epitopes.

Finally, from this base dataset, the BCPred/FBCPred group generated 10 final datasets, composed of sequence sizes: 12, 14, 16, 18, 20, 22, 24, 26, 28, 30. To create the 22, 24, 26, 28, and 30 residue sized epitopes, the 20 residue sized epitopes and non-epitopes were extended on both ends. To create the 12, 14, 16, and 18 residue sized datasets, the 20 residue sized epitopes were truncated on both ends. By creating these 10 different sized sequence length based dataset variations, the BCPred/FBCPred group was hoping to see how classification accuracy of a system changes when one changes the sliding window length. BCPred/FBCPred group made the original non homology reduced dataset, and the 10 derived datasets, available online at [46]. Because our system is also based on a sliding window method, and thus requires finding an optimal sliding window, we chose to train it using these 10 datasets.

#### Benchmark measures

Our system can be applied to residue chains of any length by utilizing a sliding window approach that moves forward one residue at a time along the chain. Once it reaches the end of the entire protein sequence, it provides a score for each residue. Thus, benchmark measurements, accuracy, and AUC, are more fine grained and are based on the correctly predicted epitope residues, rather than correctly predicted epitopes. Those predicted residues which all fall into a single continuous sequence, are considered by DRREP to form a single continuous epitope. This classification approach allows DRREP to provide smoother decision boundaries and classify variable length eptiope and non-epitope sub-sequences within some large sequence to which it is applied, as opposed to providing scores for fixed length blocks of residues. Accuracy, Sensitivity, and Specificity, are calculated as follows:

$$\begin{aligned} \textbf{Sensitivity}& = TP/(TP+FN)\\ \textbf{Specificity} &= TN/(TN+FP)\\ \textbf{Accuracy} &= (TP+TN)/(TP+FP+TN+FN)\\ \end{aligned} $$

where TP (True Positive), FP (False Positive), TN (True Negative), FN (False Negative), are residue based. The Receiver Operating Characteristic (ROC) plot is True Positive Rate (Sensitivity) vs. False Positive Rate (1-Specificity), with AUC calculated as the area under the ROC curve. AUC has been demonstrated to be highly correlated with the general performance of a classifier, with a higher AUC being correlated with a classifier capable of high sensitivity, specificity, and accuracy.

### Deep ridge regressed epitope predictor

The Deep Ridge Regressed Epitope Predictor (DRREP) is a deep neural network composed of 5 hidden layers, but only a single learning layer. The first layer is a randomly generated array of k-mers, used to perform feature extraction using basic string mismatch functions, with the mismatch number set to 0. Because the activation function just counts and outputs how many times a particular k-mer occurs in the input string, it can also be considered to be using a bagging method introduced by Leo Breiman [47]. But because each k-mer is slid across the entire input sequence, with the second neural layer performing a pooling computation, the first layer can also be considered as performing a convolutionary computation. The second layer is composed neurons which form a normalization pooling layer. The third is a layer of linear neurons, whose weights are set analytically using a simplified ridge regression algorithm [48]. The hidden weights of the linear neural layer are analytically computed using a matrix inverse, in our case, the Moore-Penrose generalized inverse, a method also used in a number of other machine learning algorithms [49–52]. This is followed by a fourth scaled average pooling layer, and then a final fifth thresholding layer. This final fifth layer is composed of a single threshold neuron whose synaptic weights are deduced by DRREP using the validation scores of the sub-networks it is composed of, acquired during the training process. These validation scores are used to weigh the sub-network contributions to the final classification score. In essence, making the DRREP function as a type of ensemble of sub-networks.

In the following subsections we discuss the Moore-Penrose generalized inverse calculated synaptic weights, followed by a pseudo-code and a detailed discussion of the entire DRREP pipeline.

#### Calculating synaptic weights analytically

DRREP can be applied to a continues sequence of any length, producing a score for each residue. The way DRREP does this internally is by using its sliding window to cut the long sequence into sub-sequences, score each subsequence, and then recompose the sub-sequences, averaging out the prediction for each subsequence such that the resulting longer sequence has a score for each residue. Thus, DRREP has a long sequence as input, and then it internally cuts it down to create a dataset of Y columns and X rows, where Y is the length of the sliding window used (chosen by the researcher during the training phase), *X*=*T* *o* *t*_*R* *e* *s* *i* *d* *u* *e* *s*−*S* *l* *i* *d* *i* *n* *g* *W* *i* *n* *d* *o* *w* *L* *e* *n* *g* *t* *h*+1, and *T* *o* *t*_*R* *e* *s* *i* *d* *u* *e* *s* is the total number of residues in the original long input sequence.

Each of these sliding window sized sub-sequences is passed through the first string function based layer and the second norm-pooling layer. The second layer outputs a matrix: **H**, which then acts as an input matrix to the third linear neural layer containing the synaptic weight matrix: *β*. During the training phase, the input data is labelled, and is usually composed of a dataset of sliding window sized sub-sequences, each of which is either an epitope or a non-epitope. Thus, we expect for the hidden linear neural layer to produce the expected training output (labels/classes) matrix: **E**, based on the available labelled input and *β*. We can calculate *β* by solving:

$$\mathbf{H}\beta = \mathbf{E} $$

where matrix **E** is composed of target labels, or in our case, epitope and non-epitope classes, and matrix **H** is composed of the output vectors of the 2nd pooling neural layer which is based on the output of the first string function neural layer that processed the labelled input vectors. The optimal weight matrix of the linear hidden neural layer, *β*, is then computed through the use of Moore-Penrose generalized inverse, all in a single step, as follows:

$$\beta = \mathbf{H}^{\dagger}\mathbf{E} $$

where **H** ^*†*^ is the Moore-Penrose generalized inverse of matrix **H**.

Because the string function based first hidden neural layer which performs the extrapolation of the input data into the feature space, is randomly generated, and because regression is performed using the Moore-Penrose generalized inverse, the algorithm is fast, and is used here akin to the way it is used in [53]. Because there is no pre-training, or long phases of iterative training as is done in the more standard approaches based on gradient descent, it opens doors to potentially training the system on *big data*.

#### Training, validation, and DRREP construction

**Fig. 1:**
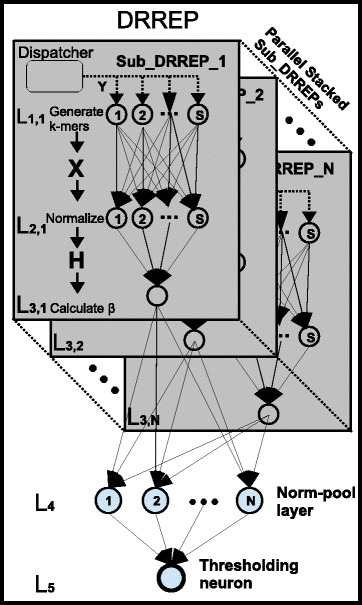
DRREP Architecture DRREP is composed of N Sub_DRREPs. Each Sub_DRREP’s first layer, L1, is composed of S neurons, each of which is made out of a randomly generated k-mer based mismatch function. The second layer, L2, is composed of S nodes, with the entire layer performing normalization of the L1’s signals. Layer L3, is the learning linear neural layer, whose synaptic weights are calculated using the Moore-Penrose generalized inverse. All N Sub_DRREPs are stacked in parallel. The L4 is a norm-pooling layer, composed of N nodes, which normalizes the signals from each Sub_DRREP. The next layer, L5, is composed of a single thresholding neuron, which weighs each contribution from the Sub_DRREPs based on that Sub_DRREP’s relative validation score, and passes this value through the threshold to output the final score for the input sliding window

DRREP is a 5 layer deep neural network based on a parallel stack of independently trained 3 layer based sub-networks, each with a single learning layer, a randomly generated string (k-mer) based activation function first hidden layer, and a pooling transformational layer, as shown in Fig. 1. Training is done in multiple phases. First, *N* number of 3 layer neural networks, called Sub_DRREPs, are generated and trained independently (each such Sub_DRREP network is composed of the DRREP’s first 3 layers). The *N* networks are then stacked in parallel, with each network’s output aggregated, and then normalized by the norm-pooling 4th layer. The normalized signals are then passed on to the threshold neuron in the 5th layer. The way this is performed, is by putting these sub-networks in parallel, to form a single, wider, deep network. Then the fourth layer is added, which normalizes and pools the outputs from these sub-networks. The fifth layer is composed of a single thresholding neuron. The scaling factor for each sub-network is based on its relative validation AUC score, which act as weights for the single thresholding neuron in the final 5th layer, which decides whether the input vector belongs to an epitope. The DRREP pipeline is shown in Fig. 1.

The Sub_DRREP networks can be trained on input sliding windows of different sizes *Y*. We have explored sliding window sizes: 12,14,16,18,20,22,24,26,28, and 30. Though DRREP can be composed of Sub_DRREPs of different sized sliding windows, in this paper we have explored composing DRREP where all Sub_DRREP networks use the same sized sliding windows. We have explored different values for *Y*, and different values for the parameter *N* (total number of Sub-DRREPs), and settled on *Y*=24 and *N*=20, which resulted in the best validation score, and a DRREP that was fast to train.

DRREP makes its predictions purely based on the amino acid sequence. The first hidden layer in each Sub_DRREP is composed of a random number *S* of basic mismatch activation functions, each of which uses a randomly generated k-mer whose size ranges between 1 and the size of the sliding window *Y*. Based on our experiments, a string mismatch activation function which allows for 0 mismatches, produces the best results. Thus, each neuron using the basic mismatch activation function in the first layer counts the number of times its k-mer occurred in the sequence of the sliding window.

This allows the second normalization layer to calculate the proportions of various types of k-mers occurring within the window. Our intuition is that there are numerous small k-mers which are particularly antigenic, but we do not know which ones, or in which order and ratios they should be to trigger an immune response. Our system generates a large number of random k-mers, and through regression the system finds the correlation between the ratio and combination of the presence of these k-mers, and antigenicity.

Through meta-parameter optimization, DRREP was found to perform best (highest Validation dataset AUC) when for each Sub_DRREP, *S* was randomly chosen between 2 and 4000 (done in 2 bouts with a randomly generated value between 1 and 2000 for each). DRREP’s sliding window moves through the long input sequence, and for each sliding window, DRREP’s basic mismatch functions in the first hidden layer output the number of times their k-mer appeared in the window. The second pooling hidden layer in DRREP normalizes these scalar values, producing a k-mer ratio vector, and then passes this vector onwards to the 3rd layer. The third layer is the learning layer, whose synaptic weights are computed in a single step after all the training input vectors (windows) have been processed by the first 2 layers to produce the matrix **H**. The synaptic weights are computed using the Moore-Penrose generalized inverse, using the provided labels for the training dataset. This is done for each Sub_DRREP independently. Their (Sub_DRREPs) outputs are then passed onwards to layer 4, where they are pooled and normalized (this time, between the Sub_DRREPs, rather than the neurons within each single Sub_DRREP as was done in the 2nd hidden layer). Finally, the 5th layer is composed of a single threshold neuron with *N* weights, one for each Sub_DRREP. After the training and validation phases for the entire DRREP are completed, the synaptic weights for this neuron are set to the validation AUC scores of each Sub_DRREP, so that the voting contribution of each Sub_DRREP is based on its performance on the validation dataset. The neuron calculates its output in the standard linear neuron fashion, through the application of the dot product, resulting in the final output score. This output score can then further be passed through a threshold, so that the output is a classification rather than a score. By default, the threshold of the neuron is set to the mean score of the entire score sequence that DRREP produces (made possible by DRREP first calculating all the scores, and then calculating the threshold based on the mean).

#### The pipeline

**Fig. 2:**
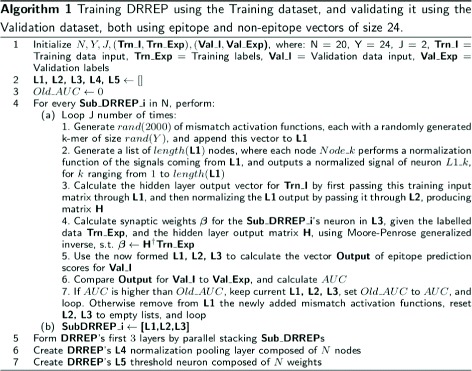
Training DRREP The figure presents the algorithm used to train DRREP

First a training dataset is 90/10 split into subsets, with 90% of the total dataset used for training, and 10% set aside to be used for validation. The training dataset is designated by the input dataset and its expected labels/classes as: (*T* *r* *n*_*I*,*T* *r* *n*_*E* *x* *p*) and validation dataset with its labels/classes as: (*V* *a* *l*_*I*,*V* *a* *l*_*E* *x* *p*). *I* and *Exp* postfixes designate **I**nput and **Exp**ected (label) matrices of the dataset. The 3rd hidden layer in each Sub_DRREP is composed and trained using the method shown in Fig. 2.

**Fig. 3:**
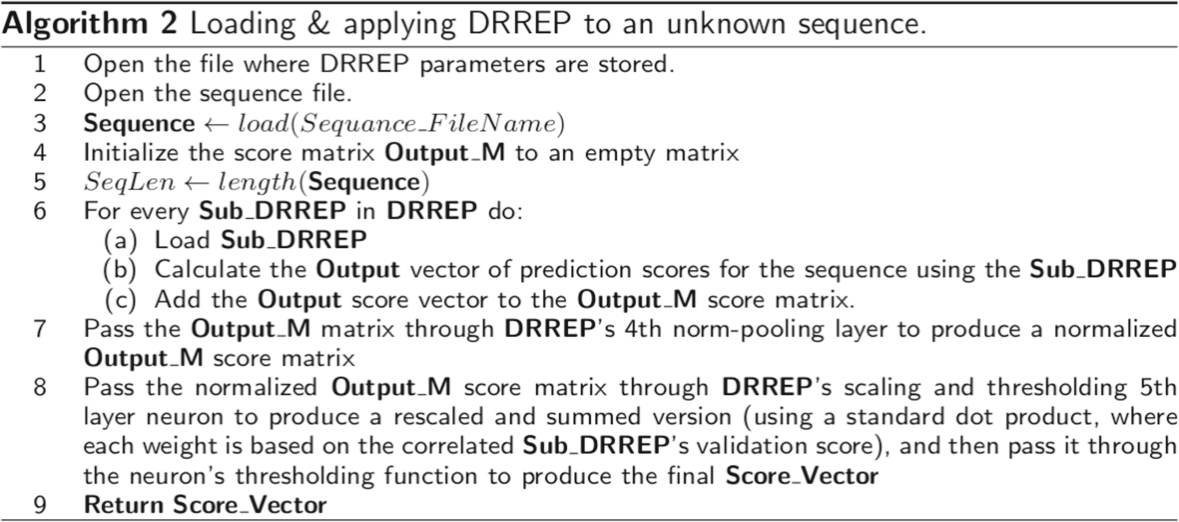
Loading and Applying DRREP The figure presents the algorithm used to load and apply DRREP

Once DRREP is trained and validated using the provided dataset, it can then be used for epitope discovery and classification by applying it to protein sequence datasets, as shown in Fig. 3.

DRREP can be updated with new Sub_DRREP networks over time, as new training data becomes available. This is done by simply stacking the new sub-networks in parallel with the existing sub-networks within the DRREP pipeline. In a similar fashion, sub-networks can also be removed if needed (exp. a Sub_DRREP is found to contribute negatively to the final prediction).

DRREP was first optimized with regards to its meta-parameters. We explored multiple sliding window sizes, and multiple first hidden layer sizes, and optimal number of Sub_DRREPs to form the DRREP. We found that sliding window of size 24, with 20 total Sub_DRREPs, each composed of around 4000 randomly generated string mismatch functions in its first layer, produced the highest validation AUC. Once the meta-parameters were optimized based on the best validation AUC score, the system was then tested by being applied to long continuous protein sequences. DRREP was implemented using JuliaLang, a high performance technical computing programming language. But because DRREP is composed of nearly 80000 first hidden layer neurons, and stored in human readable XML format, there is roughly a 40 s overhead in loading the system into memory, which is only done once.

## Results

The DRREP pipeline was applied to 5 datasets (SARS, HIV, Pellequer, AntiJen, and SEQ194) composed of long continuous protein sequences, with the AUC and accuracy at 75% specificity shown in Table 1. In the same table, we also list the AUC scores reported by other epitope predictors, such as the self reported AUC values of BCPred on the SARS dataset, BepiPred on the HIV dataset, and multiple systems on the SEQ194 dataset. Where possible, we ran the server-available predictors on the SARS and HIV datasets, these included the CBTOPE, Epitopia, ABCPred, LBtope, and DMN-LBE predictors.

**Table 1:** Accuracy and AUC results of applying DRREP to long protein sequence datasets, and the AUC results of other epitope predictors

DataSet	Tot Residues	Epitope%	System	75spec	AUC
SARS	193	63.3	**DRREP**	**86.0**	**0.862**
			BCPred	80.3	_
			ABCPred	67.9	0.648
			Epitopia	67.2	0.644
			CBTOPE	75.6	0.602
			LBtope	65.8	0.758
			DMN-LBE	59.1	0.561
HIV	2706	37.1	**DRREP**	61.4	**0.683**
			BepiPred	_	0.60
			ABCPred	61.2	0.55
			CBTOPE	60.4	0.506
			LBtope	61.2	0.627
			**DMN-LBE**	**63.6**	0.63
Pellequer	2541	37.6	**DRREP**	62.7	**0.629**
			LBtope	60.9	0.62
			**DMN-LBE**	**62.8**	0.61
AntiJen	66319	1.4	**DRREP**	73.0	**0.702**
			**LBtope**	**74.2**	**0.702**
			DMN-LBE	_	_
SEQ194	128180	6.6	**DRREP**	**75.9**	**0.732**
			Epitopia	_	0.59
			BEST10	_	0.57
			BEST16	_	0.57
			ABCPred	_	0.55
			CBTOPE	_	0.52
			COBEpro	_	0.55
			LBtope	75.3	0.71
			DMN-LBE	_	_

CBTOPE and Epitopia servers produced score based outputs, whereas ABCPred produced a list of index start locations of the predicted 16-sized window based epitopes. This required a conversion to a single residue score based format, and was performed by using the highest epitope score for each residue’s location. We have also used the CBTOPE and ABCPred servers to calculate scores for the HIV dataset. We had difficulty running Epitopia on the longer HIV dataset, as the server produced run-errors. Also, unfortunately, DMN-LBE server predicts one sequence at a time. Thus, due to the large number of sequences AntiJen and SEQ194 datasets are composed of, we were only able to run the smaller SARS, HIV, and Pellequer datasets on the server (doing so manually, one sequence at a time). For the missing predictors, or where the AUC scores are not listed in the table, we did not get a response from the authors as to the proper conversion from the output format produced by their predictor, to the score based format we needed to calculate AUC and accuracies, or the server for that predictor was not available. Nevertheless, we applied DRREP to every dataset (without retraining), so that it could be compared to every system which was originally tested on it.

**Fig. 4:**
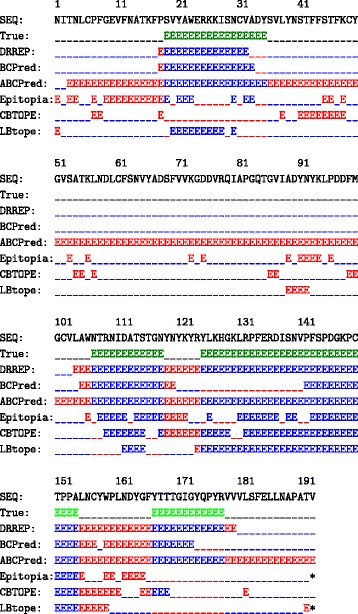
Predicting Epitopes in SARS SARS sequence (SEQ), true epitope locations (True), DRREP’s predicted epitopes (DRREP), BCPred’s predicted epitopes (BCPred), ABCPred’s predicted epitopes (ABCPred), EPitopia’s predicted epitiopes (Epitopia), CBTOPE’s predicted epitopes (CBTOPE), and LBtope’s predicted epitopes (LBTope). The incorrect predictions are colored in red, the correct are colored in blue, and the true epitope locations are colored green

Figure 4 demonstrates the type of output DRREP provides when using the classification threshold, rather than simply outputting a list of residue scores. The figure shows the SARS sequence, with the line (True) showing the actual epitope locations, line (DRREP) shows DRREP’s epitope predictions when using a 75% specificity threshold, line (BCPred) designates BCPred’s predicted epitopes, line (ABCPred) designates ABCPred’s predicted epitopes, and similarly for EPitopia, CBTOPE, and finally LBtope. The true epitope locations are coloured green, and for each predictor, the incorrect predictions are coloured in red, and the correct are coloured blue.

### Discussion

When applied to the SARS sequence, DRREP achieved an AUC of 0.862, and an accuracy of 86.0% at specificity set to 0.75. BCPred reported an accuracy of 80.3% at the same specificity. We also used the ABCPred, Epitopia, CBTOPE, LBtope, and DMN-LBE servers to generate predictions for the SARS sequence. Their resulting AUCs were 0.648, 0.644, 0.602, 0.758, and 0.561 respectively. Their accuracies calculated at a specificity set to 0.75 are also listed in the table. DRREP achieved a higher accuracy and AUC than all competing predictors on this sequence. This is an improvement in accuracy of 5.7% over BCPred, and an AUC improvement over the best performing predictor on that dataset (LBtope here, because BCPred did not report it’s AUC for this sequence) of over 13.7%.

Furthermore, from Fig. 4 we can see that DRREP predicted correctly a larger number of residues than other predictors. But, DRREP classified the four sub-sequences as all belonging to a single epitope. This could potentially be alleviated by adding a post-processing filter which calculates not just a score, but changes in a score as well. We base this hypothesis on the fact that we observed a number of cases where the score transitioned significantly between continuous sequences of epitope and non-epitope sequences, yet still held above the epitope threshold for both cases. Based on this observation, perhaps the system could be further improved by taking into account radical score transitions. This methodology is planned to be explored in our future research. From all the test datasets on which LBtope was tested, it performed the worst on SARS. For the tests performed, the version of LBtope used was based on the one trained on a fixed 20 residue window based dataset, which was used in the original LBtope publication. When using this version, the server does not predict the last residue, hence it was designated with an asterisk. An LBtope trained on flexible window based original dataset was also tested on SARS, because that version does predict the last residue, but it performed much worse than the version shown, and thus was not included. The Epitopia server also did not provide the classification for the 193rd residue in the SARS sequence. Another interesting anomaly in residue prediction results was produced by ABCPred. ABCPred server gives a score for 16 residue slices, with a default epitope score threshold set to 0.5. Based on this, ABCPred classified numerous such 16 residue slices as epitopes, and when combined together, this included all but the first two residues in the SARS sequence.

When applied to the HIV dataset, DRREP produced an AUC of 0.683. We can compare it to LBtope and DMN-LBE, which were also tested on the HIV dataset, their AUCs were 0.627 and 0.63 respectively. We ran ABCPred, BepiPred, and CBTOPE servers on the same dataset, and their resulting AUCs were 0.60, 0.55 and 0.506, respectively. Thus, DRREP achieves an AUC higher by 0.053 than the best predictor in the list (DMN-LBE), an improvement of 8.4%. Interestingly, at 75% specificity DMN-LBE had a higher accuracy.

BepiPred was trained on the Pellequer dataset, and was thus disqualified from being compared on it. We had a difficult time running this dataset on multiple predictor servers, and neither could we find their performance on this particular dataset within published literature. The only server we were able to run on the dataset was LBtope, which achieved an AUC of 0.62, which is 3.2% lower than DRREP’s AUC of 0.629. On Pellequer DMN-LBe, though having a lower AUC score, at 75% specificy achieved an accuracy of 62.8%, which was.1% higher than DRREP.

AntiJen is a dataset much larger than HIV and Pellequer, and thus we could not get it to run on some of the listed predictor servers, nor find their published performance on this dataset. The only server that allowed us to run such a large dataset was LBtope. DRREP and LBtope tied on this dataset with an AUC of 0.702. LBtope did achieve a 1.2% higher accuracy at 75% specificity.

DRREP achieved an AUC of 0.732 on the SEQ194 dataset, which we compared to Epitopia, ABCPred, CBTOPE, and COBEpro, whose AUCs on this dataset were acquired from [26], and BEST10/16 system whose AUC is listed in [24]. This dataset was also ran on the LBtope server, which achieved an AUC of 0.71. DRREP achieved an AUC performance improvement of 5.6% over the best performing predictor (LBtope). It should be noted that the dataset SEQ194 is the most recently published of all datasets, with the largest number of long, FASTA encoded sequences. Furthermore, LBtope was ran using the *LBtope_Fixed_non_redundant (non redundant dataset)* version, which was the one reported in their most recent paper, and we considered to be the best performer of the versions available.

Thus, DRREP achieved a higher AUC performance on 4 of 5 datasets than all other predictors, and particularly the state of the art LBtope and DMN-LBE predictors. DRREP tied with the LBtope on the remaining fifth dataset, the AntiJen dataset. And though DMN-LBE achieved a higher accuracy at 75% specificity on the HIV dataset, and parity on Pellequer, it will not be possible to know the 75% specificity threshold when the systems are applied to new and unknown sequences, thus AUC is still the best indicator of system’s general performance. These results demonstrate DRREP’s markedly higher general performance.

## Conclusion

In this paper we have presented a novel deep network based classifier using a string activation function based first layer, multiple non-linear transformational pooling layers, and a single learning layer. The learning layer synaptic weights are calculated analytically using the Moore-Penrose generalized inverse, making the training phase faster than that of SVM, and standard gradient descent based models. When DRREP was applied to the SARS sequence, the achieved classification accuracy at 75% specificity was 86.0%, which is 5.7% higher than the BCPred/FBCPred, it’s AUC was higher by over 13.7% than that of LBtope on the same sequence. When applied to the HIV, Pellequer, and SEQ194 datasets, DRREP achieved an AUC performance improvement of 8.4%, 3.2%, and 5.6% respectively, over the best performing predictors in the list, which were the most recently published DMN-LBE and LBtope predictors. The only dataset on which DRREP did not achieve a performance improvement was the AntiJen dataset, on which both DRREP and LBtope achieved the same AUC score. We believe that these results represent a substantial and highly regular and stable improvement over the current state of the art.

DRREP is a promising new method. Its generalization capabilities are stable across all tested datasets, with different levels of epitope densities. We plan to further improve DRREP’s performance by incorporating new advancements within the deep learning domain, by further exploring convolutional layering, local receptive field layering, and other types of topologies and pooling paradigms. We also plan to further explore the effect of training dataset refinement on the system’s performance. The DRREP system [54] and its datasets [55], are freely available on the GitHub server, and can be downloaded from the referenced URLs.
